# Identification and Potential Roles of Human MicroRNAs in Ebola Virus Infection and Disease Pathogenesis

**DOI:** 10.3390/genes15040403

**Published:** 2024-03-25

**Authors:** Melvin Mensah-Bonsu, Christopher Doss, Clay Gloster, Perpetua Muganda

**Affiliations:** 1 Applied Science and Technology Ph.D. Program, North Carolina A&T State University, Greensboro, NC 27411, USA; mmensahbonsu@aggies.ncat.edu; 2 Department of Electrical and Computer Engineering, North Carolina A&T State University, Greensboro, NC 27411, USA; cdoss@ncat.edu; 3 Department of Computer Systems Technology, North Carolina A&T State University, Greensboro, NC 27411, USA; cgloster@ncat.edu; 4 Department of Biology, North Carolina A&T State University, Greensboro, NC 27411, USA

**Keywords:** Ebola, miRNAs, small RNA-Seq, ARPE cells, pathogenic mechanisms

## Abstract

Ebola virus (EBOV) is a highly pathogenic virus that causes a severe illness called Ebola virus disease (EVD). EVD has a high mortality rate and remains a significant threat to public health. Research on EVD pathogenesis has traditionally focused on host transcriptional responses. Limited recent studies, however, have revealed some information on the significance of cellular microRNAs (miRNAs) in EBOV infection and pathogenic mechanisms, but further studies are needed. Thus, this study aimed to identify and validate additional known and novel human miRNAs in EBOV-infected adult retinal pigment epithelial (ARPE) cells and predict their potential roles in EBOV infection and pathogenic mechanisms. We analyzed previously available small RNA-Seq data obtained from ARPE cells and identified 23 upregulated and seven downregulated miRNAs in the EBOV-infected cells; these included two novel miRNAs and 17 additional known miRNAs not previously identified in ARPE cells. In addition to pathways previously identified by others, these miRNAs are associated with pathways and biological processes that include WNT, FoxO, and phosphatidylinositol signaling; these pathways were not identified in the original study. This study thus confirms and expands on the previous study using the same datasets and demonstrates further the importance of human miRNAs in the host response and EVD pathogenesis during infection.

## 1. Introduction

Ebola virus infection leads to a severe hemorrhagic fever known as Ebola virus disease (EVD), which has a high case fatality rate (CFR) [1,2]. The CFR of EVD during outbreaks can be as high as 90% [3,4]. The 2014–2016 outbreak in West Africa was the largest outbreak, resulting in a total of 28,616 cases of Ebola and 11,310 deaths reported in Guinea, Liberia, and Sierra Leone. Currently, there have been 35 recorded outbreaks in 13 countries, mostly in West and Central Africa, with the last outbreaks occurring in 2022 in D.R. Congo and Uganda [3]. Most Ebola virus outbreaks occur in remote rural areas that lack the necessary infrastructure to detect the disease early [5,6]. At present, EVD-specific therapies are limited to treating the symptoms as they arise, though advancements have been made in drug development and diagnostics [1,3]. A deeper understanding of the pathogenic mechanisms behind EVD will facilitate the development of more effective vaccines and treatments to combat the virus.

Ebola virus can efficiently target and replicate in various cell types, thereby triggering pro-inflammatory cytokines that can lead to tissue damage and impair the host’s immune system [4,7,8]. Ebola utilizes viral proteins to suppress immune responses such as interferon (IFN) response, dendritic cell (DC) maturation and T-cell stimulation, leaving the host vulnerable to viral damage [9,10,11,12]. Further studies are necessary to fully understand the pathogenic mechanisms of Ebola and develop effective treatments. Small RNAs, such as microRNAs, have shown promise in regulating gene expression and could be a promising area of research for diseases like Ebola [13,14].

The study of disease infection and pathogenic mechanisms has traditionally focused on mRNAs. A growing body of research, however, has revealed the significance of small RNAs, particularly microRNAs (miRNAs). MiRNAs, i.e., short, single-stranded RNA fragments (18–22 nt), are critical in regulating post-transcriptional gene expression during cellular processes [15]. Research has linked miRNAs to various diseases, including those caused by viral agents [15,16]. Cellular miRNAs such as hsa-miR320, hsa-miR-1246 and hsa-miR-196b-5p have been reported to inhibit EBOV’s GP-mediated cytotoxicity [17]. MiRNAs such as hsa-miR-122-5p and hsa-miR-125b-5p have also been reported as biomarkers for EVD in cynomolgus macaques [18]. EBOV has also been shown to encode its own miRNAs besides the host miRNAs expressed to regulate host and viral gene expression [19,20,21,22]. EBOV-encoded miRNA studies have demonstrated that these virus-encoded miRNAs could serve as biomarkers for early diagnosis as well as therapeutic targets [20,21]. Host miRNAs, though, have a fundamental role in gene regulation and modulation of the antiviral defense during viral infections [23]. Understanding how host miRNAs interact with viruses can therefore provide insights into the mechanisms of viral infections and potential therapeutic targets. Although research suggests miRNA involvement in Ebola disease [18,24,25], miRNA studies in this area have been limited, and the exact role of miRNAs in Ebola infection and pathogenesis is not yet fully understood.

Oliver et al. [25] demonstrated the role of miRNAs in Ebola-infected human retinal epithelial (ARPE) cells, a key component of ocular immune privilege. Ebola persists in immune-privileged sites such as the eyes, and human ARPE cells may thus serve as a reservoir for Ebola virus in the eyes [25,26]. Wang et al. [27], also observed that miR-150-3p inhibited the expression of EBOV GP and VP40 in 293T cells. There is limited research, however, on the roles that host miRNAs play in EBOV infection and pathogenic mechanisms. With continuous updates to miRNA databases, available host genomes, and miRNA discovery tools, additional and novel cellular miRNAs with a role in EBOV infection and pathogenesis can be discovered. Thus, the updated human genome assembly (T2T consortium human genome) and the current miRNA database (miRBase v 22.1) present an opportunity for this discovery.

The objective of this study was to identify and validate additional known and novel cellular miRNAs in Ebola-virus-infected cells and predict their functions. We analyzed data from a previous study by Oliver et al. [25] to identify known and novel cellular miRNAs in human ARPE cells infected with EBOV.

## 2. Materials and Methods

### 2.1. Deep Sequencing Dataset Utilized

The miRNA-Seq deep sequencing data (SRA SRP220628, BioProject: PRJNA564225) of Oliver et al. [25] were acquired from NCBI/SRA database. These data contain a total of 24 samples that include three mock-infected and three EBOV-infected ARPE cells in four replicates; nine mock-infected samples and nine Ebola-virus-infected samples were utilized for this study.

### 2.2. Differential Expression of Known Cellular miRNAs Using BWA

**Figure 1:**
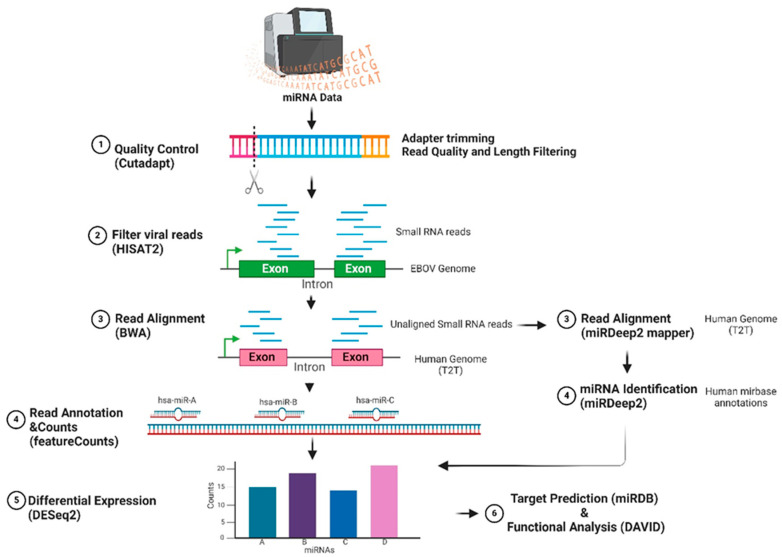
MiRNA-Seq analysis flowchart showing all the steps taken to analyze miRNA-Seq data. Created with BioRender.com.

Differential expression was conducted as described by Oliver et al., except the newer human T2T reference genome (T2T-CHM13v2.0) was utilized. The data were analyzed on a locally installed version of the Galaxy Server (Galaxy Release v 22.05) [28]. Datasets were processed through FASTQ quality control and then trimmed, and low-quality reads and adapters were removed by utilizing Cutadatpt [29], allowing for an 18 bp minimum read length. The data were filtered for EBOV reads by aligning to the EBOV genome (NCBI Reference Sequence: NC_002549.1: Zaire ebolavirus isolate Ebola virus/H.sapiens-tc/COD/1976/Yambuku-Mayinga, complete genome) using HISAT2 [30]. The unaligned reads were further processed to identify host cellular miRNAs. Burrows Wheeler Aligner (BWA) [31] was employed for alignment of the datasets to the human genome (T2T-CHM13v2.0). The featureCounts tool (version 2.0.1) [32] was used to assign miRBase annotations (v.22.1) [33] to the data. DSeq2 [34] was used to identify differentially expressed miRNAs between EBOV-infected and mock-infected samples (using the parameters *p*-value ≤ 0.05 and log2 fold-change ≥ 1) (Figure 1).

### 2.3. Differential Expression of Known and Novel Cellular miRNAs Using miRDeep2

miRDeep2 was utilized to detect known and novel cellular miRNAs expressed in the control mock and EBOV infected cell datasets. The fastq files were first processed by trimming adapter sequences and removing low-quality reads using the Trim Galore tool [35]. The pre-processed data were mapped to the human genome (T2T-CHM13v2.0) using the miRDeep2 mapper tool [36]. The miRDeep2 identification tool was then utilized to detect and identify known and novel cellular miRNAs using the human T2T genome and known human precursor and mature miRNAs from the miRBase database (Release 22.1) [33,37] as reference guides. The miRNAs identified were then analyzed for differential expression between mock-infected and EBOV-infected datasets using DESeq2. Differentially expressed miRNAs were selected using the parameters *p*-value ≤ 0.05 and log2 fold-change ≥ 1. miRNAs found in less than 50% of samples in the mock-infected or EBOV-infected groups were considered not significant.

### 2.4. Prediction of Gene Targets and Functional Analysis

The cellular miRNA targets were predicted by utilizing the custom prediction module of miRDB, a web-based server at http://mirdb.org/ (accessed on 3 January 2024) [38]. Targets with a score above 80 were selected as recommended by miRDB. The Database for Annotation, Visualization, and Integrated Discovery (DAVID) Bioinformatics Resources [39] was used for functional analysis of the regulated target genes. The KEGG pathways and gene ontology (GO) biological processes linked to the target genes were documented.

## 3. Results

### 3.1. Identification of Known Cellular miRNAs Differentially Expressed in EBOV-Infected Human RPE Cells at 24 h Post Infection

**Figure 2:**
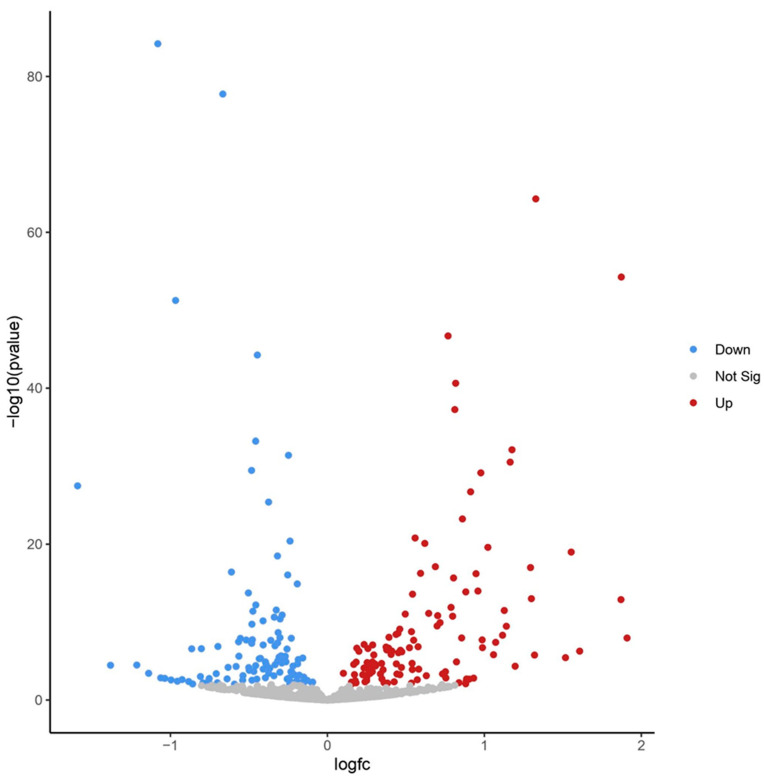
Volcano plot showing known cellular miRNAs differentially expressed in EBOV-infected human RPE cells at 24 h post infection as compared to mock-infected cells using BWA method (visual representation of Table 1).

To identify known cellular miRNAs differentially expressed in EBOV-infected human RPE cells as compared to mock-infected cells at 24 h post infection, the quality-controlled dataset was aligned to the T2T human genome using BWA, as described [25]. Using a *p*-value < 0.05 and log2 fold-change > 1, 19 miRNAs were found to be significantly increased, and 7 miRNAs were found to be significantly decreased (Table 1, Figure 2, and Table S1). Overall, under our analysis conditions, we identified 11 out of the 15 known miRNAs identified by the Oliver et al. [25] study. We also identified 15 known additional miRNAs that were not identified by the Oliver et al. [25] study.

### 3.2. miRDeep2 Identification of Known and Novel Cellular miRNAs Differentially Expressed in EBOV-Infected Human RPE Cells at 24 h Post Infection

To identify known and novel cellular miRNAs, the existing miRNA deep sequencing dataset [25] was analyzed with miRDeep2 as described in the Methods Section. Four (4) known cellular miRNAs were significantly upregulated in the two- to four-fold range within the infected cells as compared to the mock-infected cells (Table 2 and Table S2). We also identified two upregulated novel cellular miRNAs (Table 3 and Table S3). The two novel miRNAs identified were observed to be the most differentially expressed miRNAs in the ARPE cell system. NC_060925.1:203674252..203674274 was upregulated with a log2 fold change of 5.61, and NC_060929.1:151642371..151642392 was upregulated with a log2 fold change of 3.54 in the EBOV-infected cells compared to the mock-infected.

### 3.3. miRNA Gene Target Prediction and Functional Analysis

**Figure 3:**
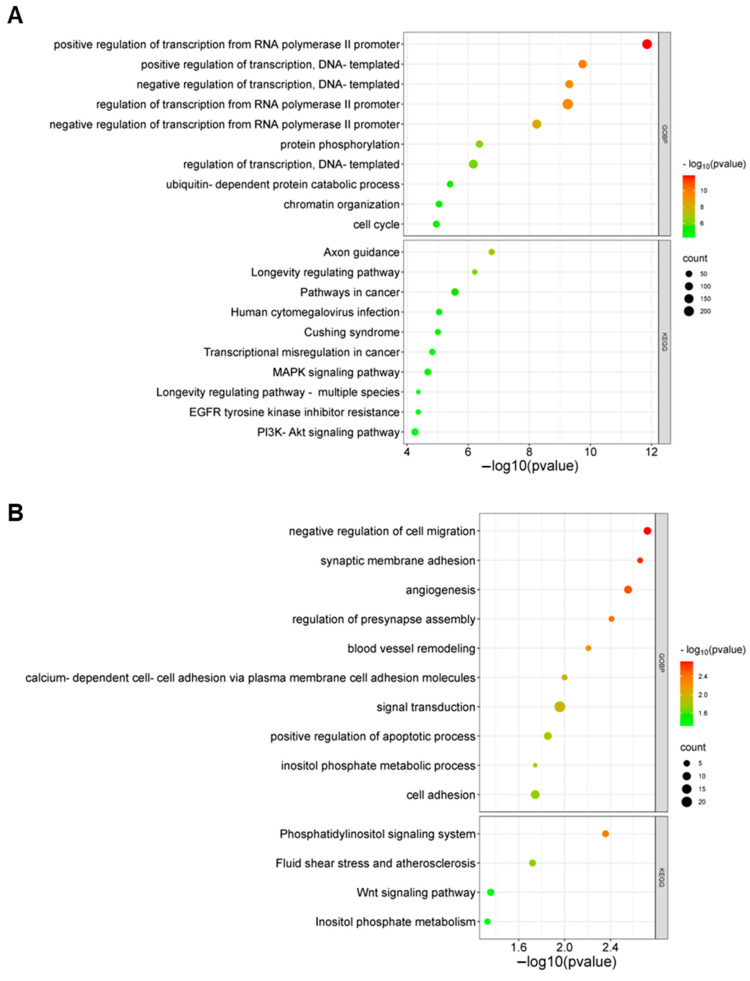
Bubble plot showing enriched KEGG pathways and biological processes of novel miRNAs identified in RPE cells from DAVID analysis: (**A**) NC_060925.1:203674252..203674274; (**B**) NC_060929.1:151642371..151642392.

**Figure 4:**
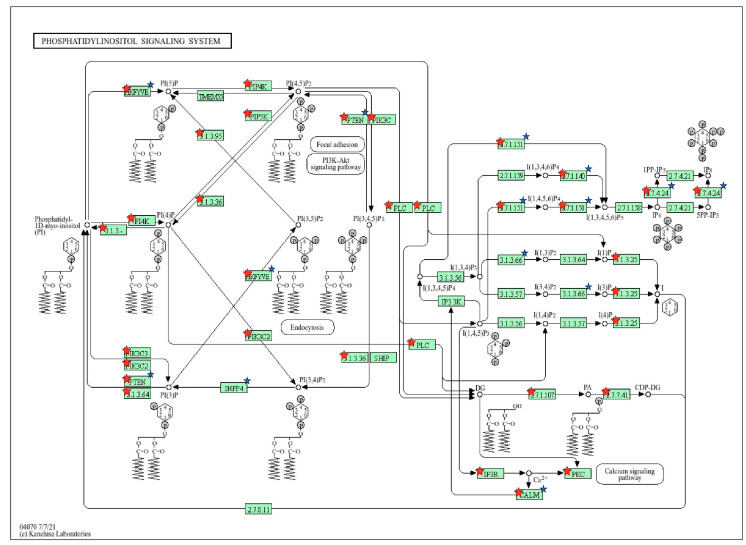
KEGG pathway of the phosphatidylinositol signaling system with target genes of our known miRNAs shown in red stars and targets of novel miRNA NC_060929.1:151642371..151642392 shown in blue stars.

**Figure 5:**
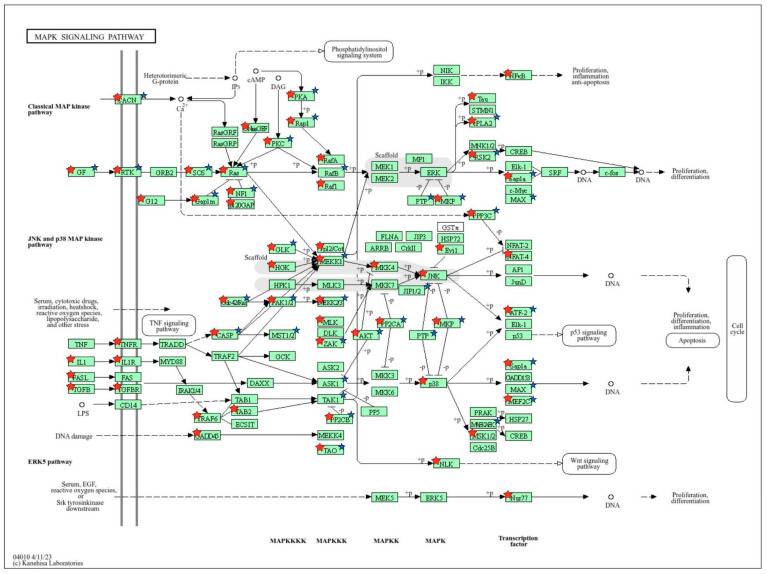
KEGG pathway of the MAPK signaling pathway with target genes of our known miRNAs shown in red stars and targets of novel miRNA NC_060925.1:203674252..203674274 shown in blue stars.

**Figure 6:**
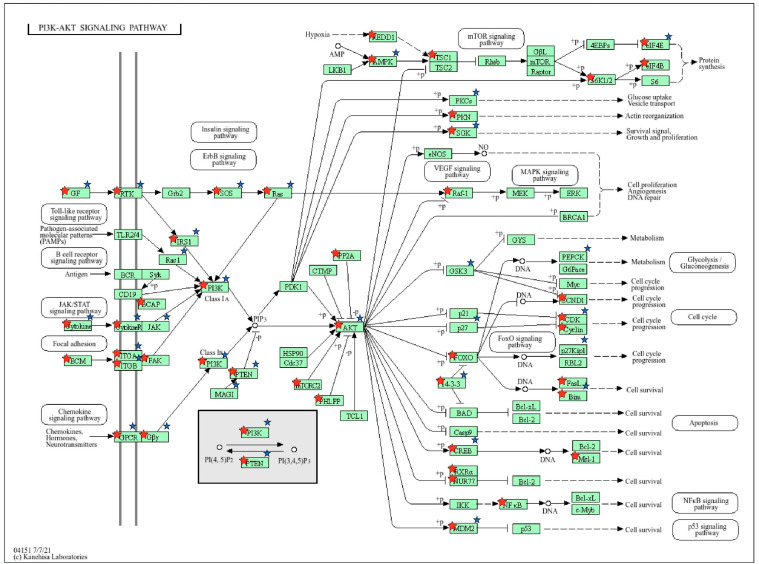
KEGG pathway of the PI3K-Akt signaling pathway with target genes of our known miRNAs shown in red stars and targets of novel miRNA NC_060925.1:203674252..203674274 shown in blue stars.

Gene targets of the differentially expressed known and novel cellular miRNAs identified were predicted using miRDB (Files S1 and S2). The predicted target genes were compared to previous gene expression data from the same dataset [26] to identify target genes with downregulated expression during EBOV infection (Files S1 and S2). Pathways and biological processes linked to the target genes of the miRNAs were identified using DAVID. The miRNAs were observed to target genes associated with pathways and biological processes that included phosphatidylinositol signaling system, PI3K-Akt signaling pathway, MAPK signaling pathway, axon guidance, transcription regulation, gene expression regulation, and protein phosphorylation (Figure 3, Figure 4, Figure 5 and Figure 6; Files S1 and S2).

## 4. Discussion

The identity and role of cellular miRNAs in EVD infection and pathogenesis are still not fully understood. Thus, our study aimed to identify additional known and novel differentially expressed cellular miRNAs in Ebola-virus-infected ARPE cells. Our study also aimed to determine the potential roles of the identified miRNAs in Ebola virus infection and disease pathogenesis. We identified 23 upregulated and 7 downregulated differentially expressed human miRNAs in the EBOV-infected ARPE cells using two miRNA identification methods. Of these, two miRNAs were novel, and an additional 17 known miRNAs were not previously identified by Oliver et al. (2019) [25] in their study. The mRNA targets of these miRNAs were associated with the following pathways and biological processes: WNT signaling, the phosphatidylinositol signaling system, the PI3K-Akt signaling pathway, Hippo signaling, the MAPK signaling pathway, Axon guidance, transcription regulation, gene expression regulation, and protein phosphorylation. Thus, this study successfully identified two novel human miRNAs as well as additional known differentially expressed human miRNAs in Ebola-virus-infected cells as compared to a previous study [25]. This study also identified potential regulatory roles of these miRNAs in EBOV infection and pathogenesis.

Our study identified 26 differentially expressed known miRNAs (19 upregulated and 7 downregulated), compared to 15 known miRNAs identified in a previous study [26], by utilizing the same dataset and BWA alignment method. Thus, an additional 11 known differentially expressed human miRNAs were identified in this study by utilizing the Oliver et al. [25] BWA method. Furthermore, the utilization of the miRDeep2 tool identified an additional four (two known and two novel) differentially expressed miRNAs not identified by the Oliver et al. [25] study. Our use of different sequence alignment strategies as well as an updated human genome and miRNA database led to our identification of a total of 13 known and 2 novel differentially expressed human miRNAs not identified by the Oliver et al. [25] study in this system. The miRDeep2 method we employed uses a two-step approach employing algorithms for identifying known and novel miRNAs based on data and features of validated precursor and mature miRNAs and a host genome [37]. In contrast, the BWA tool identifies miRNAs through sample alignment with a reference genome. The Oliver et al. [25] study also utilized the GRCh38.p3 human genome and miRBase (v.21) annotations [25]. Our study, on the other hand, utilized the latest human genome (T2T-CHM13v2.0 T2T) and the upgraded miRBase database (v.22.1). The T2T genome and the upgraded miRBase database (v.22.1) contain new information that was unavailable during the Oliver et al. [25] study. The inconsistencies also resulted in some differences in pathway prediction due to the increase in number of miRNAs identified and resultant changes in the number of mRNA targets identified.

The novel miRNA NC_060925.1:203674252..203674274 was predicted to target genes linked to Axon guidance, MAPK signaling, PI3K-Akt signaling, FoxO signaling, and Rap1 signaling. In-depth analysis revealed that this miRNA targets several genes involved in cell adhesion (ADAM22, ADGRL3, CRISPLD1, LRIG3, PCDH18, PLXDC2, and TMEFF2), which is essential for Ebola virus entry, dissemination, and host immune response [40,41,42]. Ebola binds to specific surface receptors to enter cells. Cell adhesion may be influenced in the viral–receptor interaction process through the modulation of cell adhesion and migration signaling genes and pathways [41,43]. The modulation of cell adhesion by this miRNA by targeting the above genes would impede EBOV entry, a necessary mechanism for dissemination of the virus within the host. EBOV infection also induces pro-inflammatory cytokines and chemokines and promotes interactions that directly disrupt the expression of molecules required for cell adhesion. This disruption in cell adhesion is likely to have a negative impact on immune response [40,42,44]. The activity of this miRNA in suppressing cell adhesion molecules could therefore be to the benefit or detriment of the virus. This miRNA was also found to target genes involved in the regulation of transcription, DNA repair, cell growth and differentiation, and cell signaling. EBOV has been shown to manipulate host transcriptional regulatory networks to create a favorable environment for viral replication and immune evasion [45,46]. These findings strongly suggest that NC_060925.1:203674209-203674274 could have a profound impact on Ebola virus infection and pathogenesis.

The novel miRNA NC_060929.1:151642370-151642427 was found to target genes associated with phosphatidylinositol signaling, WNT signaling, and inositol phosphate metabolism. These findings suggest that this miRNA could potentially play a crucial role in modulating viral entry and pathogenesis [47,48]. Qiu et al. (2018) [47] demonstrated that EBOV requires the production of phosphatidylinositol (3,5) bisphosphate to facilitate viral entry, and PIKfyve inhibition was found to prevent the entry of replication of EBOV. The finding that the miRNA is linked to WNT signaling is in line with reports that viruses hijack the WNT signaling pathway to modulate the cell cycle conditions allowing for the initiation and maintenance of viral pathogenesis [48,49,50]. Downregulated genes found to be targets of this miRNA include CYP1B1, which has been connected to ocular diseases such as glaucoma [51]. This observation is in line with previous studies showing the preference of the eye as a site of persistent infection and ocular problems in Ebola virus disease [25,26,52]. Based on these findings, we hypothesize that the host could increase the production of this miRNA in response to the virus’s impact on the eye.

Our findings suggest that the known miRNAs we identified are linked to various pathways, including the PI3K-Akt signaling pathway, MAPK signaling pathway, phosphatidylinositol signaling, and WNT signaling. Our findings align with previous studies that have established the association of PI3K-Akt signaling with EBOV cellular entry [53,54]. Saeed et al. [53] observed that infection by ZEBOV was significantly reduced at an early stage in replication upon inhibition of PI3K and Akt. Qiu et al. [47] also revealed the critical role of phosphatidylinositol in facilitating Ebola viral entry. We found that miRNAs including hsa-miR-32 and has-miR-1277 were significantly linked to PI3K-Akt signaling. Their interactions with genes associated with PI3K-Akt signaling would therefore suggest a role in EBOV entry inhibition during infection. MiRNA hsa-miR-32 has also been shown to be upregulated in human liver cells and cynomolgus macaques during Ebola virus infection [18,24]. Our finding that the known miRNAs identified were associated with MAPK signaling is in line with studies implicating MAPK signaling activation in Ebola virus pathogenesis by increasing viral replication [55,56]. We also observed that miRNAs were associated with pathways such as FoxO signaling and Hippo signaling, which have not been extensively studied in EVD. MiRNAs hsa-miR-32-5p, hsa-miR-1305, and hsa-miR-29b-3p were associated with FoxO signaling. FoxO signaling plays critical roles in immune responses including B-cell, T-cell, and macrophage activation [57,58]. Studies indicate that FoxO transcription factors such as FoxO1 and FoxO3 regulate immune responses to viral infections [59,60]. Specifically, FoxO1 deficiency can cause memory T cells to revert to a state of terminal differentiation, impacting secondary memory responses [61,62]. Dysregulation of FoxO signaling by these miRNAs may therefore inhibit the ability of immune cells to mount an effective response against EVD. This would suggest a pro-viral role for these miRNAs. Our research thus supports the concept that circulating miRNAs targeting these pathways may be part of the host system’s strategy to counter EBOV infection and its pathogenic mechanisms. On the other hand, EBOV could elicit the expression of some miRNAs to promote infection and pathogenesis.

Oliver et al., 2019, found that circulating human miRNAs in EBOV-infected ARPE cells were associated with pathways involving MAPK signaling, PIK-Akt signaling, cancer, and AGE-RAGE signaling pathways. Our study aligns with and expands on the Oliver et al. [25] study. We identified two novel human miRNAs and an additional 17 known miRNAs in EBOV-infected ARPE cells by reanalyzing the datasets in the Oliver et al. study. We further demonstrated the association of our identified miRNAs to pathways that include WNT signaling, FoxO signaling, and phosphatidylinositol signaling; these pathways were not identified in the Oliver et al. [25] study. The findings of this study thus expand on the previous Oliver et al. [25] study and demonstrate further the importance of cellular miRNAs in the host response and EVD pathogenesis during infection.

Our study focused specifically on host miRNA expression, though EBOV has also been shown to encode its own miRNAs [63]. EBOV-encoded pre-miRNAs and mature miRNAs have been identified, such as miR-MAY-251, miR-MAK-403, miR-T1-5p, and miR-VP-3p [20,64]. These miRNAs target human host genes involved in processes such as viral replication, hemorrhaging, and immune modulation. The regulatory effects of some EBOV miRNAs on selected host mRNA targets have been validated by studies using techniques like RT-qPCR and luciferase assays [64,65]. Studies suggest that EBOV-encoded miRNAs can be used as early diagnostic biomarkers for Ebola virus disease [63,64]. Both host and EBOV-encoded miRNAs, therefore, play essential roles in Ebola pathogenesis. Though this study does not include EBOV-encoded miRNA experiments, future studies will examine them in this cellular system.

The mRNAs and pathways targeted by the identified miRNAs can be leveraged to develop potential therapeutic strategies for Ebola disease. This information can be effectively used to identify potential biomarkers for early detection and therapeutic targets, as has been previously reported [19,20,66,67]. MiRNA profiling in Ebola-vaccine-receiving patients has identified miRNAs including hsa-miR-126-5p, hsa-miR-146-5p, hsa-miR-136, and hsa-miR-448 as potential early diagnostic biomarkers [66,67]. The knowledge gained from this study and others like this can also be used in vaccine development, creation of miRNA mimics and inhibitors, and gene therapy. Specific miRNAs can be incorporated into anti-viral drug and vaccine designs to improve their efficacy. Targeting miRNAs involved in immune cell differentiation and function could make it possible to boost the efficacy of vaccines targeting EBOV [68,69]. Our understanding of how specific miRNAs regulate pathways in Ebola pathogenesis such as viral replication, immune evasion, and cell death is very important. This understanding could lead to the development of miRNA-based therapeutics that restore the normal function of these pathways, ultimately aiding in the treatment of Ebola virus disease.

MiRNA-based therapies, though promising, come with several associated challenges. The complexity of miRNA–mRNA interactions makes it difficult to target specific mRNAs. miRNAs usually have multiple targets, and their application can lead to off-target effects, which could cause unintended consequences [70,71]. miRNAs can also be unstable and prone to degradation and may therefore have poor uptake [72]. Some mechanisms for miRNA delivery, for example, the use of viral vectors, could trigger an immune response, leading to the clearance of the therapeutics introduced [70]. Further in-depth investigation into the roles of miRNAs in Ebola is therefore needed to identify potential therapeutic targets and effective ways of delivering these therapeutics.

In summary, this study identified novel and additional known human miRNAs expressed in Ebola-infected ARPE cells as well as their predicted target mRNAs and associated pathways. Our findings provide insight on the potential role miRNAs may play in EVD. This report contributes towards our understanding of human miRNAs in Ebola virus infection and pathogenic mechanisms. Our study limitations are the same as those of the original study by Oliver et al. [25]. The study limitations include the cell type used, which limits its applicability to other cell types. Also, the data were collected at only 24 h post infection, which may limit our results to applications in early infection or early survivorship. The data were obtained from cells infected with a virulent EBOV strain; thus, the same observations may not be obtained using less virulent EBOV strains. Future in vitro studies will be necessary to validate the findings of this study. Further research in this field will aid in identifying biomarkers of Ebola virus disease and potentially lead to new therapeutic targets.

## Figures and Tables

**Table 1:** Known cellular miRNAs differentially expressed in EBOV-infected human RPE cells at 24 h post infection as compared to mock-infected cells using the BWA method.

GeneID	log2 (FC)	*p*-Value
hsa-miR-1296-3p	1.91	1.10 × 10^−8^
hsa-miR-29b-3p *	1.87	5.47 × 10^−55^
hsa-miR-33a-5p *	1.87	1.29 × 10^−13^
hsa-miR-155-3p	1.61	5.33 × 10^−7^
hsa-miR-1307-5p *	1.55	1.03 × 10^−19^
hsa-miR-548a-3p	1.52	3.65 × 10^−6^
hsa-miR-100-3p *	1.33	5.14 × 10^−65^
hsa-miR-19a-5p	1.32	1.70 × 10^−6^
hsa-miR-32-5p *	1.30	9.81 × 10^−14^
hsa-miR-33b-3p	1.29	9.98 × 10^−18^
hsa-miR-592	1.20	4.59 × 10^−5^
hsa-miR-7-5p	1.18	7.58 × 10^−33^
hsa-miR-4521 *	1.16	2.95 × 10^−31^
hsa-miR-33b-5p *	1.14	3.41 × 10^−10^
hsa-miR-1305 *	1.13	3.22 × 10^−12^
hsa-miR-1277-5p	1.12	4.88 × 10^−9^
hsa-miR-365a-5p *	1.07	3.98 × 10^−8^
hsa-miR-16-1-3p	1.06	1.53 × 10^−6^
hsa-miR-101-5p	1.02	2.53 × 10^−20^
hsa-miR-10392-5p	−1.04	1.67 × 10^−3^
hsa-miR-6735-3p	−1.06	1.43 × 10^−3^
hsa-miR-27b-5p *	−1.08	6.33 × 10^−85^
hsa-miR-1291	−1.14	3.71 × 10^−4^
hsa-let-7c-3p	−1.21	3.22 × 10^−5^
hsa-miR-3917	−1.38	3.46 × 10^−5^
hsa-miR-3074-3p *	−1.59	3.23 × 10^−28^

Differentially expressed miRNAs were selected at *p* ≤ 0.05 and log2 fold change ≥ 1. * Known human miRNAs identified by the Oliver et al. [25] study.

**Table 2:** Known cellular miRNAs differentially expressed in EBOV-infected human RPE cells at 24 h post infection as compared to mock-infected cells.

GeneID	log2 (FC)	*p*-Value
hsa-miR-29b-2-5p	1.80	3.98 × 10^−51^
hsa-miR-29b-1-5p	1.79	3.69 × 10^−65^
hsa-miR-33b-5p	1.31	5.05 × 10^−25^
hsa-miR-1305	1.06	2.30 × 10^−11^

Differentially expressed miRNAs were selected at *p* ≤ 0.05 and log2 fold change ≥ 1.

**Table 3:** Novel cellular miRNAs differentially expressed in EBOV-infected human RPE cells at 24 h post infection as compared to mock-infected cells.

GeneID	log2 (FC)	*p*-Value	Sequence
NC_060925.1:203674252..203674274	5.61	1.76 × 10^−5^	aaaugagaaaggcugucgugacu
NC_060929.1:151642371..151642392	3.54	1.39 × 10^−3^	caaaaauuguaauuacuuuggc

Differentially expressed miRNAs were selected at *p* ≤ 0.05 and log2 fold change ≥ 1.

## Data Availability

The data generated/analyzed during the current study are available in the Supplementary Files. Raw data files analyzed were obtained from the NCBI/SRA database (SRA dataset SRP220628, BioProject: PRJNA564225).

## Supplementary Materials

The following supporting information can be downloaded at: https://www.mdpi.com/article/10.3390/genes15040403/s1, Table S1: Differentially Expressed Known miRNAs identified with BWA; Table S2: Differentially Expressed Known miRNAs identified with miRDeep2; Table S3: Differentially Expressed Novel miRNAs identified with miRDeep2; File S1: Predicted Targets & Functional Analysis of miRDeep2 Identified miRNAs; File S2: Predicted Targets & Functional Analysis of BWA Identified miRNA.
